# Training Rats Using Water Rewards Without Water Restriction

**DOI:** 10.3389/fnbeh.2018.00084

**Published:** 2018-05-03

**Authors:** Pamela Reinagel

**Affiliations:** Section of Neurobiology, Division of Biological Sciences, University of California, San Diego, La Jolla, CA, United States

**Keywords:** operant conditioning, water restriction, rat behavior, high-throughput behavior, rodent training

## Abstract

High-throughput behavioral training of rodents has been a transformative development for systems neuroscience. Water or food restriction is typically required to motivate task engagement. We hypothesized a gap between physiological water need and hedonic water satiety that could be leveraged to train rats for water rewards without water restriction. We show that when Citric Acid (CA) is added to water, female rats drink less, yet consume enough to maintain long term health. With 24 h/day access to a visual task with water rewards, rats with *ad lib* CA water performed 84% ± 18% as many trials as in the same task under water restriction. In 2-h daily sessions, rats with *ad lib* CA water performed 68% ± 13% as many trials as under water restriction. Using reward sizes <25 μl, rats with *ad lib* CA performed 804 ± 285 trials/day in live-in sessions or 364 ± 82 trials/day in limited duration daily sessions. The safety of CA water amendment was previously shown for male rats, and the gap between water need and satiety was similar to what we observed in females. Therefore, it is likely that this method will generalize to male rats, though this remains to be shown. We conclude that at least in some contexts rats can be trained using water rewards without water restriction, benefitting both animal welfare and scientific productivity.

## Introduction

Operant conditioning tasks with food or water rewards are commonly used to train and test rodents in a wide variety of sensory, motor and cognitive tasks. Water rewards can be dispensed with temporal and quantitative precision and consumed rapidly with minimal body movement, which makes them ideal for automated behavioral training and testing and electrophysiological or optical recording. Thus many operant conditioning paradigms for rodents use water rewards (Skinner, 1936) including many recent studies (Bermejo et al., 1996; Keller et al., 2000; Uchida and Mainen, 2003; Abraham et al., 2004; Rinberg and Gelperin, 2006; Hromádka and Zador, 2007; Wolfe et al., 2008; Yang et al., 2008; Harvey et al., 2009; Zoccolan et al., 2009; Andermann et al., 2010; Voikar et al., 2010; Busse et al., 2011; Endo et al., 2011; Erlich et al., 2011; Meier et al., 2011; Raposo et al., 2012; Vermaercke and Op de Beeck, 2012; Poddar et al., 2013; Rodgers and DeWeese, 2014; de Hoz and Nelken, 2014; He et al., 2015; Kurylo et al., 2015; Wekselblatt et al., 2016; Francis and Kanold, 2017; Nikbakht et al., 2018).

A challenge of this approach is the need for water restriction in order to motivate animals to perform tasks. Live-in automated training systems eliminate the need for daily human intervention for training purposes. But water restriction requires a high level of monitoring, documentation and intervention by research staff, above and beyond the scope of routine animal husbandry. If it were possible to train rodents without water or food restriction, behavioral training or testing in live-in systems could be provided in the context of routine daily health monitoring by animal husbandry staff, which could increase experimental throughput. Rodents will do trials for nutritive rewards (sugar water, juice, soy milk, peanut oil, etc.) without food or water restriction, but caloric rewards require more cleaning and maintenance of equipment and increase the risk of diabetes or obesity in the animals.

Previous studies had demonstrated that rats provided with unpalatable water will drink less than when provided with plain water, but nevertheless maintain weight and general health for long periods of time. This was shown for adult male Wistar rats with 0.1% quinine hydrochloride in their water (Nicolaidis and Rowland, 1975) and for adult male Long-Evans rats with up to 4% citric acid (CA) in their water (Watson et al., 1986). In both cases rats consumed approximately half as much water when it was adulterated, and also reduced food intake. Nevertheless, weights were stably maintained at >90% control body weights. This observation suggested to us that rats maintained on *ad lib* unpalatable water would be motivated to perform tasks for water rewards. With quinine water, adaptive increases in water consumption in response to stressors (such as elevated temperature or salt) are attenuated; behavioral regulation of fluid intake was more normal with citric acid (Watson et al., 1986; Watson and Swartwood, 1990). Moreover, long term intake of quinine, as for malaria treatment, is associated with toxicity, including auditory, visual and neurological pathology (Bateman and Dyson, 1986). Therefore, citric acid is a better candidate for chronic water amendment. Previous studies of citric acid water amendment tested only young adult male rats, however. Data were lacking for female rats, juveniles, or geriatric animals.

## Materials and Methods

### Animal Care

Sixteen female Long-Evans rats (Harlan Laboratories, Indianapolis, IN, USA) were used in this study. Male rats will be studied in a future cohort. All procedures and experiments were performed in an AAALAC accredited facility, in strict accordance with international, federal and state laws, policies and guidelines, with the permission and oversight of the UCSD Institutional Animal Care and Use Committee (IACUC protocol #S04135).

**Figure 1:**
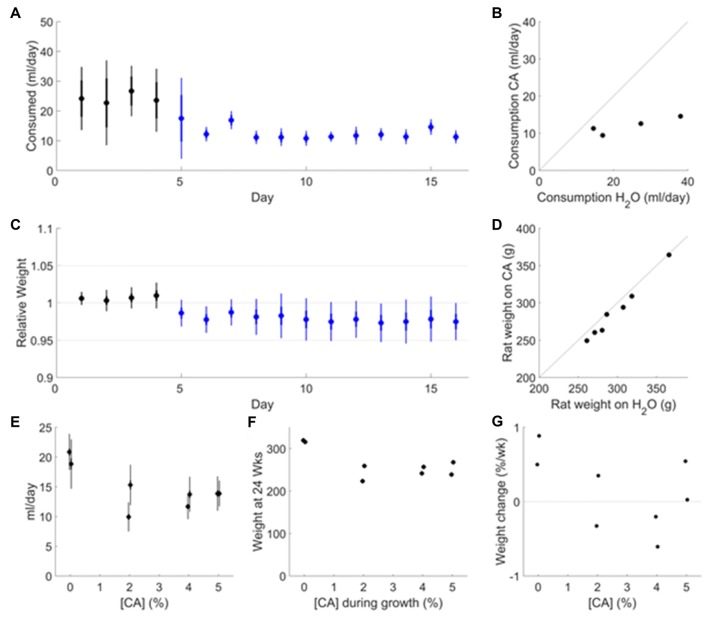
Effect of citric acid (CA) adulteration on water consumption, weight maintenance and growth.** (A)** Fluid consumption of adult rats (age ~12 months, *N* = 7) transitioning to 2% CA water after >2 weeks on plain water. Average consumption (ml per rat) is based on daily measurement of consumption per cage (pair housed). Data are shown for the last 5 days on plain water (black symbols) and for the first 12 days after switching to 2% CA (blue symbols). Error bars indicate SD (thin) and SEM (thick) over cages.** (B)** The average daily fluid consumption in each cage in the last 5 days on plain water is compared to the consumption in the same cage in the last 5 days on CA water. **(C)** Weight measured daily in *N* = 7 rats over the same time period shown in **(A)**. **(D)**The average weight of each rat in the last 5 days on plain water is compared to the last 5 days on CA water. **(E)** Daily water consumption as a function of citric acid concentration. Data are shown from a 21-day period around 6 months of age (*N* = 2 rats per condition), after maintenance on CA since P28. Error bars indicate the SD (thin) or SEM (thick) over days. These values are consistent with the daily consumption from P72–P168 as measured per cage (pair housed; data not shown). **(F)** Adult weight at 24 weeks, as a function of citric acid concentration in *ad lib* water supply during growth. **(G)** Weight change of rats over the 21 days shown in **(E)**, expressed as a percentage of weight at the beginning of that period.

Rats were housed in standard rat cages with wood shaving bedding, in a single-species single-gender room maintained at 68–72°F, ambient humidity (~50%), with a reversed light cycle. Diet was *ad lib* rat chow (Envigo Diet, 7012), which contains 0.3% sodium and 19.1% crude protein. Food consumption was not measured. An extended dark period (14 h:10 h dark:light) was used to reduce animal stress (Dulcis et al., 2013). For enrichment, in addition to the task itself, all animals were supplied with wood block chew toys and PVC tubes, and were pair housed except during live-in training. A single piece of cereal (LIFE, Quaker Oats Co., Ravenna, OH, USA) was given to each rat during daily weighing. Baby carrots (5–10 grams/day) were given to support growth of juvenile rats that were water restricted, except for rats being raised on CA water for Figures 1E,F. Citric acid water was mixed fresh weekly using food-grade citric acid (BulkSupplements or Milliard), at concentrations of 1%–5% (weight/volume) in distilled de-ionized water. *Ad lib* (plain or citric acid) water was provided in standard glass water bottles changed at least weekly, and daily* ad lib* consumption was monitored by weighing the bottles.

Rats are prandial drinkers and thus simultaneous availability of food and water promotes both food consumption and fluid consumption in water-restricted animals. During live-in training, rats were singly housed in standard rat cages attached to training chambers by a connector tube, such that rats could freely move between the training chamber (where water rewards are delivered) and the living chamber (where food, and *ad lib* CA water if applicable, are provided). For the 2-h training protocol, rats were removed from pair-housed living cages into separate training chambers during sessions. Naïve animals were confined to the training chamber, in which case rat chow was placed within the chamber. Once rats reliably engaged the task, the training chambers were attached to housing cages as per the live-in protocol.

Water-restricted rats, including those with 24-h task access, as well as all rats with citric acid in their water, were weighed daily and assessed for other clinical signs of dehydration. No rat showed clinical signs of dehydration during this study. Any rat weighing <90% of reference weight was by policy given *ad lib* plain water until normal weight was restored. This occurred in two experiments in which rewards were smaller than intended and the rats were otherwise water restricted.

### Long-Term Maintenance With CA in Water

For the experiment of Figures 1A–D, seven adult female Long-Evans rats (six in pair-housed cages and one single housed) were transitioned from *ad lib* plain water to *ad lib* CA water. Fluid consumption was measured daily per cage, and body weight was recorded daily for each animal. For the experiment in Figures 1E–G six female Long-Evans rats in pair-housed cages were transitioned from *ad lib* plain water to *ad lib* CA water at age P28. For acclimation, CA concentration was increased from P28–P72 gradually as tolerated from 1% CA up to the final concentration of 2%, 4% or 5%. Two control rats were maintained on 0% CA (plain water) in parallel. Fluid consumption was measured daily per cage, and body weight was recorded daily for each animal. The CA concentrations were then held constant from P72–P168 to monitor long-term fluid consumption and growth rate. No other source of fluids was provided.

### Training in Visual Task

Eight female Long-Evans rats were trained under our standard water restriction protocol until they were expert subjects in a visual task. The apparatus and general training paradigm (Meier et al., 2011) and visual task (Petruno et al., 2013; Reinagel, 2013b) were as previously described. Briefly, training and testing were controlled with custom software written in Matlab (Mathworks, Natick, MA, USA). Visual stimuli were displayed on a computer monitor using the Psychophysics Toolbox (Brainard, 1997; Pelli, 1997). Rats initiated trials or indicated responses by licking one of three water ports, which we detected using infrared emitter-detector pairs. Liquid rewards were dispensed by opening solenoid valves attached to a pressurized water supply. The software automatically progresses through a series of shaping steps according to performance criteria (Meier et al., 2011).

**Figure 2:**
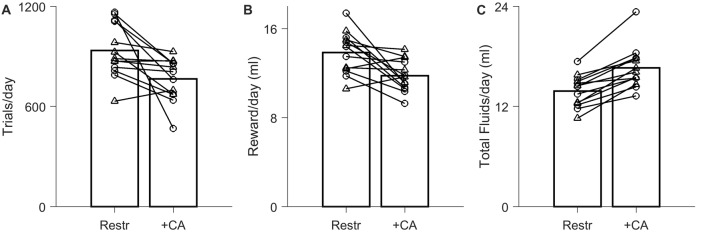
Effect of *ad lib* CA water on motivation to work for water rewards in a live-in testing protocol. Data from *N* = 13 experiments on *N* = 7 rats. The *ad lib* water contained either 2% CA (circles, *N* = 7) or 4% CA (triangles, *N* = 6). **(A)** Average trial rate with 24 h/day task access, when the only source of water was task rewards (Restr) vs. when *ad lib* CA water was also present 24 h/day (+CA). Bars indicate population averages; lines indicate the *N* = 13 paired comparisons. **(B)** Total rewards earned per day in the water-restricted condition vs. *ad lib* CA condition. **(C)** Total fluid intake (sum of earned rewards and *ad lib* CA water consumption).

**Figure 3:**
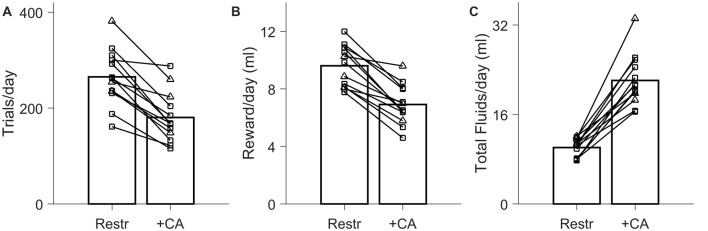
Effect of *ad lib* CA water on trial rates in 2-h daily sessions. Data from *N* = 13 experiments on *N* = 7 rats. In all panels, circles indicate comparisons in which no supplemental water was given in either condition (*N* = 10); triangles indicate cases in which supplemental water was required and provided in the restricted condition (*N* = 3). **(A)** Number of trials performed per day in 2-h daily testing sessions, when rats were water restricted the other 22 h (Restr) vs. when rats had *ad lib* 2% CA the other 22 h (+CA). **(B)** Quantity of water earned in the form of task rewards, with and without *ad lib* 2% CA between sessions. **(C)** Total fluid intake per day (sum of earned water rewards, *ad lib* 2% CA consumption, and supplemental water if applicable).

**Figure 4:**
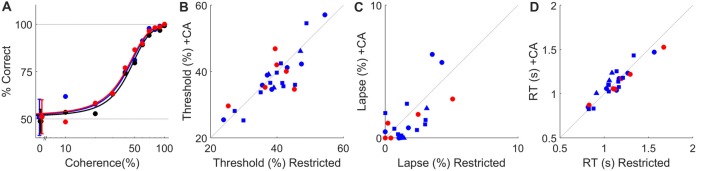
*Ad lib* CA water does not impact psychophysical performance. **(A)** Psychometric curves for a typical rat tested under water restriction (black) or with *ad lib* water containing 2% CA (blue) or 4% CA (red) in the live-in protocol. Dashed lines indicate chance and perfect performance. Psychophysical thresholds (defined as coherence at which accuracy is 75%) were 43%, 41% and 40% coherence for restricted, 2% CA and 4% CA respectively. Lapse (error rate at 100% coherence) was ≤1% in all three conditions. In panels **(B–D)**, data from 24-h task access are plotted as circles, with color indicating CA concentration (blue for 2%, red for 4%). Data from 2-h task access are plotted as squares (no supplement) or triangles (supplemental water in restricted condition), as in Figure 3. **(B)** Psychophysical threshold (% coherence at 75% correct) with water restriction vs. with *ad lib* CA water in all *N* = 26 experiments from Figures 2, 3. **(C)** Psychophysical lapse with water restriction vs. with *ad lib* CA water. Data shown on this scale are for the *N* = 23 experiments with lapse <10%. In three other experiments lapse was >10% on both conditions (off scale); these were also not affected significantly by *ad lib* CA. **(D)** Mean reaction time with water restriction vs. with *ad lib* CA water.

**Figure 5:**
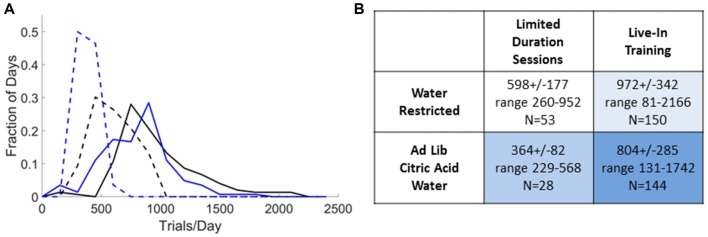
Trial rates achieved in practice.** (A)** Distribution of trial rates obtained over all sessions that used small rewards (<25 μl) for *N* = 10 expert rats, excluding the first day after switching from one training condition to another. For limited-duration sessions (dashed lines) we included all sessions 2–6 h in duration preceded by >12 h off task. The qualifying sessions had durations of 2.8 ± 0.6 h (Restricted, black) or 2.4 ± 0.3 h (with CA, blue). For live in training (solid lines) we included all sessions 18–30 h in duration preceded by <6 h off task. The qualifying sessions had durations of 22.8 ± 2.4 h (Restricted, black) or 23.5 ± 3.1 h (with CA, blue). **(B)** Summary statistics of distributions in **(A)**. Shading indicates 24 h/day access to water requiring task engagement (pale blue), from a water bottle (medium blue) or both (dark blue).

Age P30 rats began shaping with a 2-alternative forced choice (2AFC) target localization task, on which they exceeded a trial rate of 200 trials per day in 7 days on average (range 4–18 days) and exceeded 80% correct by the 17th day of training on average (range 7th–38th day). Rats were then put on a random dot motion direction discrimination task with 80% coherence, on which exceeded 80% correct after an average of 16 days (11–24) on that task. All eight rats learned both the target localization and random dot motion tasks to criterion by day 50 of training (<P80). We then varied coherence of random dot motion from 0% to 100% to measure psychometric threshold and lapse, for a period of 24 days on average (12–40) before placing rats on the final testing task (0%–100% coherence with a larger number of smaller dots). Transition to the final task occurred before age P100 in all cases (total training days 38–67, average 59). On average performance was 76 ± 8% correct (live-in) or 77 ± 6% correct (2-h sessions). Rats were tested daily on this task for several months prior to the experiments described in Figures 2–4. During some of this time the home cages were connected to the task chambers 24 h a day, such that rats had continuous access to water rewards in the visual task. At other times, rats were restricted 22 h/day and tested for 2 h/day (see Figure 5).

During training, the stations were supplied from a common pressurized water supply, and reward volume estimated based on calibration of the water valves. For more accurate measurement of reward volumes during testing, we used 50 ml syringes as gravity water supplies to the individual stations, and recorded the change in syringe water level over each session. This method eliminated variation in reward size due to fluctuation in water pressure and allowed us to measure and account for the fact that when rats lick the water tube they partly occlude flow, such that less water was delivered than predicted from calibration with unobstructed flow.

### Testing in 24-h Task Protocol

The eight previously trained rats were removed from the task and given *ad lib* water for 6 days to establish a free water baseline weight for the experiment in Figure 2. They were then acclimated to citric acid water over 7 days and divided into single housing cages for testing. Reward volume was set to ~20 μl for all rats, which generally provides a good compromise between high trial rates and adequate hydration in our live-in, water restricted protocol. Rats were tested for 2 weeks on the task with *ad lib* 2% CA, 2 weeks with *ad lib* 2% CA water alone, 2 weeks on the task with water restriction, and again for 2 weeks on *ad lib* 2% CA water alone. The order of these testing conditions was balanced across rats. We monitored task performance (trials/day, accuracy and reaction time), body weight, consumption of task reward water, and *ad lib* CA consumption daily. Rats were then acclimated to 4% *ad lib* CA water and the entire 8-week experiment repeated using *ad lib* 4% CA. From the measured reward consumption, we determined that in the *N* = 13 experiments contributing to Figure 2, the reward volumes were 20.1 ± 3.7 μl. Three other experiments were excluded from this analysis because the empirically measured reward size was found to be much less than intended (<12 μl).

### Testing in 2-h Daily Sessions

For testing the effect of *ad lib* CA on 2-h daily sessions (Figure 3), the eight previously trained expert rats in paired housing were either water restricted or given *ad lib* 2% CA. If restricted, the rats were placed on the task 2 h/day the following day, and daily thereafter with no days off. If placed on *ad lib* 2% CA, rats were monitored daily until body weight and fluid consumption stabilized, and then placed on task daily with no days off. Reward size was set to ~50 μl for all rats which generally provides a good compromise between high trial rates and adequate hydration in our daily 2-h session, water restricted protocol. We monitored task performance (trials/day, accuracy and reaction time), body weight, consumption of task reward water, and *ad lib* CA consumption between sessions if applicable. Training sessions were run at precisely the same time daily, in the middle of the dark cycle (11 AM–1 PM), 7 days/week, so rats could anticipate when plain water rewards would be available. Rats had access to a standard housing cage with litter and food during the training sessions, but *ad lib* CA water was provided in the home cage only between training sessions. We report the trial rates after allowing 2–4 days to stabilize after a change in condition. Each rat was tested in two separate experiments. From the measured reward consumption, we determined that the reward sizes we achieved in practice were 49.5 ± 13.6 μl in the *N* = 13 experiments reported in Figure 3. Three other experiments were excluded from analysis because the achieved reward size was found to be much less than intended (<20 μl).

## Results

### Citric Acid Amendment of Water Is Well Tolerated by Rats

Citric acid (CA) makes water taste sour but has negligible nutritional value and no known toxicity. To determine if rats would consume enough water to maintain long-term health when adulterated with CA, adult rats (*N* = 7, age 12 months) were monitored while on *ad lib* plain water and upon transition to *ad lib* 2% CA. The rats consumed 24.2 ± 10.7 ml per day when water was unadulterated (Figure 1A, black). After transitioning to 2% CA (Figure 1A, blue), *ad lib* fluid consumption fell to 12.0 ± 2.1 ml per day, consistent with our expectation that CA makes water less palatable to rats. The amount of CA water consumed was much less variable across cages than the amount of unadulterated water consumed (Figure 1B). The rats’ weights stabilized on CA water at 97 ± 2% of their weight on free water (Figures 1C,D). This is consistent with our expectation that rats consume the amount of water they need, even if it is mildly unpalatable. We conclude that addition of 2% CA to the water has negligible effects on body weight and is innocuous for healthy adult rats.

As a more stringent test, we maintained eight female Long-Evans rats from age P28 with different concentrations of CA in their *ad lib* water supply, and monitored fluid consumption, weight and general health daily until 6 months of age. No water rewards or other sources of fluids were offered. Rats with 2%–5% CA in their water consumed significantly less water per day than controls with plain water (*P* = 4.12 × 10^−3^, Figure 1E). The amount of water consumed was not correlated with CA concentration (*P* > 0.1). All rats with CA in their water remained bright, alert and responsive and showed no clinical signs of dehydration for the duration of the study (to age 6 months). We conclude that the amount of fluids consumed when water contained CA is sufficient for normal development and long-term health of rats.

Juveniles with CA in their water had a reduced growth rate, however. Therefore, rats maintained since P28 exclusively on CA water had a significantly lower adult weight than controls (*P* = 1.26e–03; Figure 1F). Adult weight was not significantly correlated with CA concentration (*P* > 0.1) but was highly correlated with water consumption (*R* = 0.95, *P* = 2.3 × 10^−4^). At 6 months of age, the residual growth rate in control rats was <1% of body weight per week (Figure 1G). Rats with CA water had no growth on average (range −0.6% to +0.5%); the difference between CA and plain water was nonsignificant (*P* > 0.1). Among rats with CA, residual growth rate at 6 months was not correlated with CA concentration (*P* > 0.1) but was significantly correlated with daily consumption (*P* = 4.33e–02). We conclude that addition of 2%–5% CA to the water supply reduces growth rate but is otherwise innocuous for juvenile rats.

### Willingness to Work for Water Rewards

The gap between the amount of water rats need to maintain health (12–13 ml/day) and their satiety for water (20–24 ml/day) affords an opportunity to meet physiological water needs with *ad lib* unpalatable water, and still motivate performance of tasks for plain water rewards. To test whether rats would be motivated to perform trials for water rewards despite availability of *ad lib* 2% CA water, we used a different group of rats that had been trained from age P30 using automated shaping with water rewards and water restriction (Meier et al., 2011) to asymptotic performance on a self-paced random dot motion discrimination task (Newsome et al., 1989; Petruno et al., 2013; Reinagel, 2013b). We then tested the effect on task motivation of providing *ad lib* CA water to these rats.

#### Live-in Testing Condition

Our behavioral paradigm is designed for live-in, closed-economy, fully automated training, in which rats have access to the task 24 h/day and no other water supplements (Meier et al., 2011). When the previously trained rats were tested in this condition using rewards of 20 ± 4 μl, they performed 936 ± 162 trials/day (Figure 2A, “Restr”). Holding all other task parameters constant, introduction of *ad lib* 2% or 4% CA water reduced trial rates to 766 ± 129 trials/day, or 84% ± 18% of the water-restricted trial rate (Figure 2A; *P* = 1.12 × 10^−2^ by paired-sample *t*-test, *N* = 13). The total amount of reward harvested is directly proportional to trial number, and thus necessarily declined by a comparable amount, from 13.9 ± 1.9 ml/day under restriction to 11.8 ± 1.4 ml/day with *ad lib* CA water (Figure 2B). This decline in reward consumption was more than offset by the amount of CA water consumed, however, such that total fluid intake was greater (Figure 2C). Total fluid intake of rats with *ad lib* CA water was 16.6 ± 2.5 ml/day, compared to 13.9 ± 1.9 ml/day under restriction (1.2 ± 0.1 fold greater; *P* = 1.35 × 10^−5^, *N* = 13). The increase in fluid intake was independent of CA concentration.

Most rats exceeded 600 trials per day even in the presence of *ad lib* CA water (Figure 2A, +CA). When the *ad lib* water contained 2% CA, trial rates were 74% ± 17% of the restricted condition, which was significantly different from restricted (*P* = 1.52 × 10^−2^, *N* = 7). When 4% citric acid was used, trial rates were 95% ± 12% of the restricted condition, which was not significantly different from water restriction (*P* > 0.1, *N* = 6 by paired sample *t*-test).

We conclude that in the context of a live-in task, the reduction in trial number due to introduction of *ad lib* CA water is moderate and would be acceptable in many research contexts. In addition to eliminating the risk of accidental dehydration under water restriction, providing *ad lib* CA water increased the total fluid intake to above the amount required for maintenance, and closer to the amount consumed of *ad lib* plain water.

#### Limited-Duration Testing Condition

Although live-in training and testing is highly effective, it is not always practicable. Limited-duration daily sessions are more typical in systems neuroscience research, including most of our published studies (Clark et al., 2011; Meier et al., 2011; Petruno et al., 2013; Reinagel, 2013a,b). Therefore, we also tested rats in 2-h daily testing sessions, 7 days a week, with water restriction between sessions and reward size of 49 ± 14 μl. A larger reward size was chosen because it typically provides adequate hydration in 2-h daily sessions without water supplements. On this protocol the rats performed 265 ± 58 trials/day under restriction (Figure 3A, “Restr”) earning 9.6 ± 1.4 ml/day in task rewards (Figure 3B, “Restr”).

When *ad lib* CA water was made available during non-testing hours, trial rate decreased to 68% ± 13% of the trial rate in the matched water-restricted control condition (Figure 3A; *N* = 13, *P* = 8.9 × 10^−6^). The volume of reward earned declined comparably, from 9.6 ± 1.4 ml/day when restricted to 6.9 ± 1.4 ml/day with *ad lib* CA (Figure 3B; 72% ± 11%, *N* = 13; *P* = 2.3 × 10^−6^). Nevertheless, the range of trial rates in the two conditions overlapped considerably. Rats performed an average 180 ± 52 trials/day when CA water was available, compared to 265 ± 58 trials/day with water restriction. In every case the rat performed >100 trials/day even with *ad lib* CA water.

In *N* = 10 experiments, no additional water supplements were needed to maintain weight in the restricted condition; for these rats, trial rate with *ad lib* CA water was 66% ± 13% of the water restricted trial rate. In *N* = 3 of the cases, supplemental water (3–4 ml/day on average) was required for the rats to maintain weight under water restriction. In those cases, trial rate with *ad lib* CA water was 73% ± 13%, and water supplements were no longer required when *ad lib* CA water was available.

Including any water supplements that were provided, total fluid intake on the 2-h session water restricted protocol was 10.1 ± 1.6 ml/day (Figure 3C, “Restr”). This is in rough agreement with the amount of fluid we found to be sufficient for weight maintenance on the basis of CA water consumption (Figure 1). Total fluid intake was much higher when CA water was available in addition (22.1 ± 4.5 ml/day). In paired comparisons, total fluid intake was 2.2 ± 0.4 times that under restriction (Figure 2C; *P* = 2.3 × 10^−7^, *N* = 13), with no significant effect of CA concentration. Total fluid intake in the presence of *ad lib* CA in the 2-h session protocol was comparable to the daily consumption observed for *ad lib* water (Figure 1); it is unclear why it was higher than the total consumption observed with *ad lib* CA water on the live-in protocol (16.6 ± 2.5 ml/day, Figure 2C).

We conclude that using a limited daily session protocol, trial rates with *ad lib* 2% CA were within a factor of two of those attained with water restriction holding all other trial parameters constant. If supplemental water is otherwise given to maintain weight under water restriction, introduction of *ad lib* CA water is more nearly neutral with respect to task motivation. In addition to eliminating the need for manually administered supplemental water and the risk of accidental dehydration, *ad lib* CA water increased total daily fluid intake to indistinguishable from *ad lib* water consumption.

#### Reward Devaluation Does Not Alter Task Performance

In both the limited-duration session and live-in protocols, rats consumed CA water to meet part of their total fluid requirements. To this extent the task reward was devalued. This could have resulted in less motivated behavior and therefore inferior task performance in the trials that were performed. This does not appear to be the case, however. In the visual task we used, psychometric curves were indistinguishable with or without *ad lib* CA water (e.g., Figure 4A). Perceptual threshold (Figure 4B), lapse (Figure 4C) and mean reaction time (Figure 4D) varied widely between animals, but none of these performance measures were significantly changed by the addition of *ad lib* CA water.

#### Trial Rates Achieved in Practice

In general, we strive to strike a balance between achieving high trial rates and ensuring stable weight maintenance with minimal intervention. In 2-h daily sessions, we can get higher trial rates than reported above by using smaller reward sizes. But then the rats often need water supplements and sometimes need to be removed from the task, which is disruptive and labor intensive. Instead we choose a more conservative reward size that tends to reliably keep all rats at weight and on task without supplements or expert trainer supervision.

To provide a realistic indication of the highest trial rates achieved in practice in our hands, we surveyed the historical session data for *N* = 10 rats and report the trial rates achieved in all sessions that used rewards of <25 μl (*N* = 375 sessions), as a function of the training schedule and restriction condition (Figure 5). Even when reward sizes are matched, limited-duration sessions had lower trial rates than live-in sessions: 62% if restricted or 45% with CA water. *Ad lib* CA reduced the trial rate to 61% (limited-duration training) or 83% (live-in training) of the trial rate with water restriction. With *ad lib* CA and 2-h sessions, trial rates were the lowest of the four protocols, yet 364 ± 82 trials/day might be adequate for some studies. Smaller rewards resulted in higher trial rates in the limited-duration sessions, compared to Figure 3. With water restriction, rats in limited-duration sessions with small rewards required supplemental water after 26% of the training sessions (4.14 ± 1.66 ml/day), however. No water supplements were required for weight maintenance on any of the other three training protocols.

## Discussion

### Implications for Efficiency of Experiments

We have shown that providing *ad lib* CA water can obviate the need for water restriction for behavioral tasks that depend on water rewards. In the context of a 24 h/day training protocol, the reduction in trial rate is minimal, while eliminating water restriction can dramatically reduce the amount of intervention required and therefore increase the throughput of rodent training. In an in-cage automated training system access to water is continuous, but dependent on computers and custom electronic components. Thus, water access could be interrupted in case of power failure or equipment malfunction. It is also dependent on the animals’ willingness to engage the task; even animals with continuous access can fail to perform enough trials to meet their hydration needs with water rewards. Therefore daily attention from research personnel remains essential to preclude accidental dehydration. If training staff are not on duty weekends and holidays, animals must be placed on *ad lib* water, which significantly reduces the number of training days per month. By contrast, animals with *ad lib* CA water can be maintained by standard animal husbandry procedures. Animal care staff can perform routine daily health monitoring and replace water bottles exactly as for non-restricted animals, except that the water bottles contain 2% CA. With a live-in task animals can therefore train or test 365 days a year, with scientific personnel physically examining the animals and checking equipment once a week, otherwise monitoring training progress remotely. This is achieved without providing a caloric reward that could in the long-term increase risk of complications such as diabetes or obesity.

Live-in training and testing on this protocol can be fully sufficient for data collection for some studies, such as quantitative study of perception or cognition in intact animals as a function of task parameters, or measuring the effects of biological variables that act on long time scales (e.g., mutants, disease models, lesions, or stem cell implantations). For other studies it is important for animals to perform a large number of trials in a short time interval, such as during electrophysiological recording or optical imaging, or acute chemogenetic or optogenetic manipulations. For this situation, we show that testing with *ad lib* CA is possible, but higher trial rates can be achieved in limited-duration sessions with water restriction. Such studies are more likely to benefit from a hybrid approach. Animals can fluidly switch between live-in and limited-session schedules and between water restricted and *ad lib* CA conditions (Figures 2–5). We routinely train and test animals on a live-in basis with *ad lib* CA, and still obtain high trial rates in limited-duration sessions on the first or second day after starting water restriction (unpublished observations). Therefore, studies that require long shaping sequences or extensive practice to master complex behavioral tasks could benefit from live-in training of animals with *ad lib* CA, followed by short-duration water-restricted sessions in which data are collected.

In some cases, all training and testing must be done in short duration sessions, for example if there is no live-in version of the task, or there are insufficient apparatus to devote one to every animal. In this case, the advantage of eliminating water restriction is less obvious, because scientific personnel must physically interact with the animals daily for training regardless. Depending on the demands of the study, ~360 trials/day obtained with *ad lib* CA may be acceptable, however, and the reduction in trial rates may be offset by the benefit that weight maintenance becomes reliable and effortless. With water restriction, the cognitive load of monitoring for weight loss and providing individualized water supplements or task adjustments can be the limiting factor for the number of animals one trainer can manage.

Even if water restriction is required to obtain higher trial rates, it may be advantageous to give *ad lib* CA instead of *ad lib* plain water on weekends or other days off, so that training can be resumed the first day personnel return. Otherwise animals would not perform trials at all until the following day, after restriction was re-established.

For some studies, however, *ad lib* CA is not appropriate and water restriction remains strictly necessary for other scientific reasons, for example when the scientific research question or manipulation is specifically related to reward valuation or taste processing.

### Implications for Animal Health and Welfare

By all clinical assessments, long-term amendment of the water supply with CA was physiologically innocuous for both juveniles and adults (Figure 1). We noted that rats with 5% CA in their water showed behavioral signs of stress (skittishness, aggression). We did not observe any evident stress or depression in rats with lower concentrations of CA in the water. We have the most experience with 2% CA, with which we observe that rats are active, curious and non-aggressive, play with cage-mates and toys, and eagerly accept food treats, well into advanced age (22–28 months). Later post-mortem necropsy found no evidence of health complications related to life-long CA consumption (*N* = 14 rats, data not shown). We note that in many animal housing facilities water is routinely acidified to inhibit bacterial growth. This acidification is considered an animal welfare measure and is believed to be benign for healthy adult rats and mice, although it should be considered a significant experimental variable (Les, 1968; Hall et al., 1980; Tober-Meyer et al., 1981; Hermann et al., 1982).

We did observe a reduced growth rate in juveniles raised on CA water, which was directly related to their reduced fluid consumption. Based on previous research this is likely due to reduced food consumption (Watson et al., 1986). For the purpose of this demonstration, these rats were not given access to water rewards in tasks nor any other source of hydration. Under ordinary conditions, juvenile animals would have been training in a task with water rewards, which our data show would have greatly increased their daily fluid intake and therefore their growth rate. Water supplements, days off, or hydrating treats (carrots) would also normally be available as additional means to promote normal growth rates in juveniles. Therefore the reduced growth rate we report is a worst-case scenario, and easily remedied.

In adult mice, 0.9% CA in *ad lib* water was found to have no significant effect on weight gain, body fat content, lipid profiles, glucose homeostasis, or adipose tissue inflammation, although rats with 1.5% sucrose in their water had impaired glucose tolerance and elevated inflammatory cytokines compared to sucrose alone (Leandro et al., 2016). Acidification of water affects the gut microbiome (Hall et al., 1980), which in the context a Type I Diabetes model is protective against pathology (Wolf et al., 2014). These findings support the overall safety of CA amendment of drinking water, while also pointing out that interactions with other experimental variables (such as dietary modifications and disease models) must always be considered.

Although properly monitored animals maintain weight and long-term health on water restricted protocols as well, it is preferable to avoid water restriction whenever alternatives are available. Our results show that providing *ad lib* CA in addition to water rewards is not only safe, but beneficial to animals compared to the alternative of water restriction. It increases net fluid consumption and eliminates the risk of accidental dehydration.

### Potential for Further Optimization and Extension

Increasing trial rates could broaden the contexts in which *ad lib* CA could be used instead of water restriction. Generally, rats will perform more trials if reward size is smaller. Optimization of reward size can be a subtle art, however. Under water restriction, if reward size is too small rats fail to maintain weight and therefore training cannot be sustained without frequent interventions such as giving supplemental water or changing reward size. With *ad lib* CA, if reward size is too small the rats drink more CA water, thereby making up fluid deficits and maintaining weight without human intervention (our unpublished observations). This failure mode only impacts experimental yield, without placing animals at risk of accidental dehydration. Therefore, in the presence of *ad lib* CA it would be entirely safe for an automated system to dynamically change the reward size to systematically optimize trial rates for each rat and task.

Although all rats in this study (*N* = 16) tolerated 2% CA well, we found that not all would maintain weight consistently with 4% CA (data not shown). For this reason, we selected 2% CA as our standard water amendment, including the experiment of Figure 3. In general, the lowest concentration of CA sufficient to achieve the necessary trial rate should be preferred, as long as qualitative task performance is not impaired. Our consumption data suggest that rats might also work for water rewards with 1% CA in the *ad lib* water supply. Our data indicate, however, that trial rates might be lower. On the other hand, if trial rates are insufficient with 2% CA, it is possible that trials could be increased by using for each rat the highest concentration of CA that it will tolerate in the sense of adequate fluid intake, stable weight maintenance and overall health.

In the past we have tried providing *ad lib* plain water and using palatable liquid rewards (50% soy milk or 2 mg/ml saccharine) to motivate task performance without water restriction. This approach had some success but was not reliable or effective enough for routine use. In combination with *ad lib* CA water, however, using palatable liquid rewards could increase trial rates above what we observed with plain water rewards. Non-nutritive sweeteners have the advantage that they are not perishable and do not alter the nutritional intake of animals from the established health-optimal diet.

These experiments were done with female Long-Evans rats. The keys to the success of the method is that rats drink less citric acid water than their satiety for plain water (thus maintaining motivation for the task), but enough to maintain long-term health (thus, are not water restricted). These facts have already been demonstrated for male Long-Evans rats (Watson et al., 1986). It is likely that the methods described here can be adapted to other rat strains and other rodent species as well, but this remains to be demonstrated.

## Author Contributions

PR conceived of the study, designed the experiments, analyzed the data and wrote the manuscript.

## Conflict of Interest Statement

The author declares that the research was conducted in the absence of any commercial or financial relationships that could be construed as a potential conflict of interest.
